# Process evaluation for the delivery of a water, sanitation, and hygiene mobile health program: randomized controlled trial of the WASHmobile PICHA7 program

**DOI:** 10.1186/s41182-025-00881-8

**Published:** 2026-01-07

**Authors:** Presence Sanvura, Kelly Endres, Jean-Claude Bisimwa, Jamie Perin, Cirhuza Cikomola, Justin Bengehya, Ghislain Maheshe, Raissa Boroto, Alain Mwishingo, Lucien Bisimwa, Camille Williams, Christine Marie George

**Affiliations:** 1 Center for Tropical Diseases and Global Health, Université Catholique de Bukavu, B.P 265, Bukavu, Democratic Republic of the Congo; 2 Department of International Health, Johns Hopkins Bloomberg School of Public Health, Baltimore, MD 21205-2103 USA; 3 Faculty of Medicine, Université Catholique de Bukavu, B.P 265, Bukavu, Democratic Republic of the Congo; 4 Bureau de L’Information Sanitaire, Surveillance Epidémiologique et Recherche Scientifique, Division Provinciale de La Santé/Sud Kivu, Ministère de la Santé Publique, Hygiène et Prévention, B.P 265, Bukavu, Democratic Republic of the Congo

**Keywords:** Mobile health, Cholera, Severe diarrhea, Behavior change

## Abstract

**Background:**

Diarrhea outbreaks including cholera have reached global highs this year. In the Democratic Republic of the Congo (DRC), there are estimated to be over 93 million diarrhea episodes annually. Effective and scalable water, sanitation, and hygiene (WASH) interventions are urgently needed to reduce diarrheal diseases in the DRC. Mobile health (mHealth) reminders have been shown to reduce disease morbidity and increase health-protective behaviors. Therefore, WASH mHealth programs present a promising approach to improve WASH behaviors.

**Methods:**

The WASHmobile Preventative-Intervention-for-Cholera-for-7-days (PICHA7) program is a targeted WASH intervention combining voice and SMS mHealth messages and quarterly in-person visits delivered to diarrhea patient households in DRC to reduce diarrheal diseases. During the randomized controlled trial of WASHmobile, 1196 participants received weekly WASHmobile program voice, Interactive Voice Response (IVR) quiz, and text messages over 12 months. Outcome indicators included % of unique voice, IVR, and text messages received (fidelity) and % of unique messages fully listened to (dose), assessed using the engageSPARK mobile message platform, and program reach to households assessed through monthly follow-up visits.

**Results:**

Eighty-four percent of households received unique text messages and 90% of unique voice and IVR messages were answered. Households reported receiving a WASHmobile mHealth message in the past 2 weeks at 72% of surveillance visits (844/1177). Seventy-four percent (309/418) of participants reported sharing a WASHmobile mHealth message with another person at least once.

**Conclusion:**

These findings show high fidelity, dose, and reach of mobile message delivery in the WASHmobile mHealth program. This study demonstrates the feasibility of delivering the WASHmobile PICHA7 program in eastern DRC and provides important insights for delivering WASH mHealth programing in low- and middle-income countries globally.

**Trial Registration:**

NCT05166850.

**Supplementary Information:**

The online version contains supplementary material available at 10.1186/s41182-025-00881-8.

## Background

Diarrhea outbreaks have reached global highs this year with cholera outbreaks in 32 countries [1]. There are an estimated 2.9 million cases of cholera annually resulting in over 95,000 deaths worldwide [2, 3]. The Democratic Republic of the Congo (DRC) has one of the highest rates of cholera in Africa [4]. Previous studies have identified consuming untreated water, stored drinking water being uncovered, using an unimproved water source, lack of handwashing with soap, and consuming food outside one’s home as significant cholera risk factors [5, 6]. Household members of cholera patients are at a 100 times higher risk of cholera infections compared to the general population during the 7-day period after the cholera patient is admitted to the healthcare facility [7, 8]. Furthermore, this high risk of diarrheal disease transmission to the household members of diarrhea patients spans beyond cholera to other enteric diseases including patients with *Shigella* and enterotoxigenic *E. coli* [9, 10].

Water, sanitation, and hygiene (WASH) interventions have the potential to reduce cholera and severe diarrhea globally. A previous study in the DRC found that delivery of a hygiene kit with soap and a handwashing station to suspected cholera patient households resulted in a 56% lower incidence of suspected cholera (defined by diarrhea, vomiting, or healthcare facility visit for diarrhea) [11]. Furthermore, our previous randomized controlled trial (RCT) in Bangladesh found that delivery of the targeted Cholera-Hospital-Based-Intervention-for-7-Days (CHoBI7) program, which included a health promoter visit in the healthcare facility and a similar hygiene kit, resulted in a significant reduction in bacterial culture confirmed cholera [12]. However, major challenges remain in identifying scalable approaches to promote sustained WASH behavior change over time for high-risk populations for cholera.

Mobile health (mHealth) presents a scalable approach to deliver health communication programs at a low cost, which has been shown to increase disease prevention behaviors [13–15]. To develop a targeted WASH mHealth program for high-risk populations for diarrhea, our research group designed WASHmobile, a mHealth program that promotes WASH behaviors among diarrhea patient households and those at high risk for diarrhea through voice and text mobile messages sent from a doctor at a local diarrhea hospital. This program started in Bangladesh with the WASHmobile CHoBI7 mHealth program, with weekly automated voice, Interactive Voice Response (IVR), and text messages sent from “Dr. Chobi” to diarrhea patient households for 12 months, combined with a single in-person healthcare facility visit during the time of treatment for the diarrhea patient [16]. The RCT of the WASHmobile CHoBI7 mHealth program found this WASH intervention significantly increased handwashing with soap, improved stored drinking water quality, and reduced diarrhea prevalence and stunting in diarrhea patient households [17]. In India, a WASH mHealth program for new mothers also observed increases in handwashing with soap behaviors [18]. Similarly, in Kenya a mHealth pilot program that sent weekly messages four times a week for 3 months on diarrhea symptoms, causes, and prevention measures to mothers found that participants had higher rates of handwashing with soap and water treatment behaviors compared to a control group, though these measures were self-reported [19]. These findings suggest that mHealth is a promising approach to increase WASH behaviors and reduce diarrheal diseases. However, to date, no RCTs of a WASH mHealth program have been conducted in sub-Saharan Africa.

To address this gap, in partnership with the Ministry of Health in the DRC our research group developed the WASHmobile Preventative-Intervention-for-Cholera-for-7-days (PICHA7) mHealth program, a WASH mHealth messaging system for diarrhea patient households, as a country-specific adaptation of the broader WASHmobile Program. The WASHmobile PICHA7 program delivers automated weekly voice, IVR, and text messages promoting handwashing with soap, water treatment, and safe water storage from “Dr. Picha” a doctor at a local cholera treatment center, with messages sent over a 12-month period using the web-based engageSPARK platform. Our recent RCT of the WASHmobile PICHA7 program (2334 participants) found that the program significantly increased handwashing with soap and water treatment relative to free chlorine, improved stored drinking water quality relative to *E. coli*, and reduced both healthcare facility visits for diarrhea and stunting in young children [20]. The objective of this present study was to conduct a process evaluation of the WASHmobile PICHA7 program to assess the fidelity, dose, and reach of the mHealth program components during our recently completed RCT.

## Methods

### Study design

We evaluated the WASHmobile PICHA7 program from December 2021 to December 2023 in a two-arm cluster-RCT (where a cluster is defined as a diarrhea patient household) in Bukavu city in South Kivu province of eastern DRC with a follow-up period of 12 months. Bukavu is an urban city with a population of over one million that experiences frequent conflict [20].

The target population for this study was diarrhea patients and their household members. Daily diarrhea patient surveillance and recruitment was conducted at 115 public and private health facilities from December 2021 to December 2022. Eligible diarrhea patients met the following criteria: (1) diarrhea patient admitted to a health facility with three or more loose stools over a 24 h period; (2) provided a blood and a stool sample within 24 h of enrollment; (3) had a child under five years old living in the household; (4) planned to reside in Bukavu for at least 12 months; (5) resided at home for the three nights before hospitalization; (6) no functioning tap to collect water inside the home (mostly informal settlements); and (7) at least one working mobile phone in the home. If the diarrhea patient was eligible and willing to participate in the WASHmobile PICHA7 program, both they and their household members were enrolled and, as a household (cluster level), were randomly assigned to a study arm. Randomization (1:1) of diarrhea patients to study arms was conducted using a random number generator, with assignments carried out by the study biostatistician (JP) using R (version 3.3.0). Household members were eligible if they planned to reside in the same household as the index diarrhea patient for the next 12 months, shared meals from the same cooking pot, and had resided in the same home with the diarrhea patient for the past three days. Additional details on the study design and recruitment are reported elsewhere [20].

Participants were enrolled in one of two study arms: the standard message arm, where participants received the standard discharge recommendations for diarrhea patients in the DRC on oral rehydration solution (ORS) use for rehydration, or the WASHmobile arm, where participants received the standard arm plus the WASHmobile mHealth program.

WASHmobile PICHA7 program delivery combines in-person visits with a mHealth program. The WASHmobile program is healthcare facility initiated, with a health promoter delivering a WASH module in the healthcare facility to diarrhea patients and their accompanying household members, together with a hygiene kit, which includes a water vessel with lid and tap, chlorine tablets, a handwashing station, and soapy water bottle (water bottle with small pieces of bar soap inside, a low-cost alternative to bar soap [21]). Households also received quarterly home visits for 12 months, where health promoters deliver tailored WASH messages with a focus on handwashing with soap, safe drinking water storage, and water treatment with chlorine tablets.

**Fig. 1:**
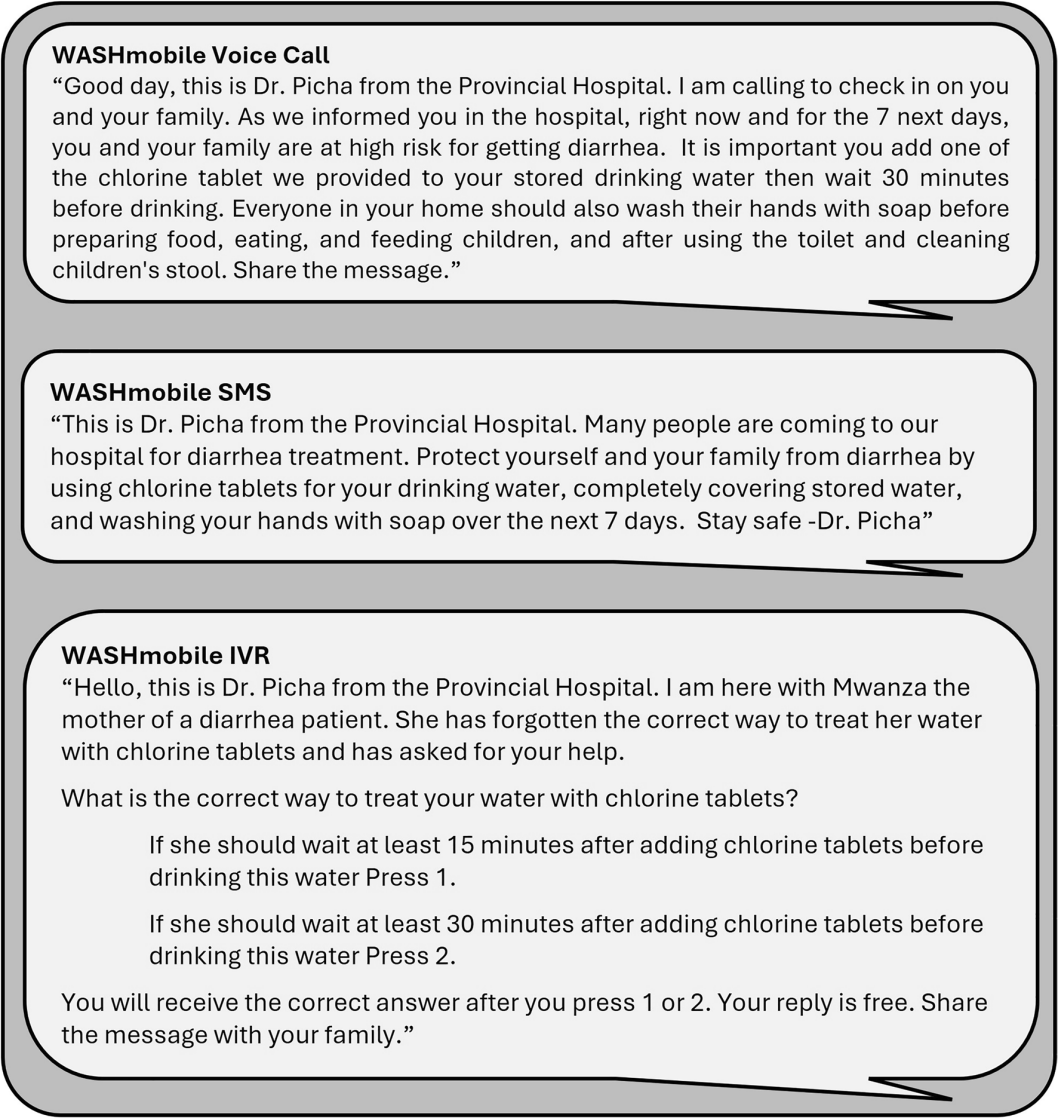
Example WASHmobile PICHA7 Interactive Voice Response (IVR), voice, and text (SMS) messages

WASHmobile PICHA7 is then reinforced through a mHealth program with weekly automated IVR, voice, and text messages focused on key WASH behaviors. Every week, each household receives one voice and one IVR message, each accompanied by a summary SMS message. A total of 55 unique voice, 55 unique IVR, and 110 unique summary text messages were sent during the 12-month program period. Figure 1 shows an example IVR, voice, and text message. All IVR, voice, and text messages sent are provided in Additional File 1. Two characters deliver the PICHA7 mHealth messages: “Dr. Picha” and “Mwanza”. Dr Picha is a doctor at a cholera treatment center who calls and texts participants to share information and reminders on handwashing with soap and water treatment behaviors. Mwanza is a woman who brought her young child to the healthcare facility for diarrhea treatment and learned proper handwashing with soap and water treatment behaviors from Dr. Picha. Dr. Picha is played by RB, a physician on our research team who provides care to diarrhea patients at a cholera treatment center. RB reviewed the content of messages and recorded the automated messages sent to participants.

Subscription to the WASHmobile PICHA7 mHealth program was performed by a health promoter who entered a shortcode into the phone of the diarrhea patient and their household members at the healthcare facility. To train diarrhea patients and their household members on receiving and responding to WASHmobile mHealth messages, the health promoter sent a practice voice, IVR, and text message to the mobile phones of the household members of patients present in the healthcare facility. They also provided support with technical challenges encountered when answering practice calls, responding to practice IVR messages, and opening text practice messages. The web-based engageSPARK platform was used to send WASHmobile  mHealth program messages. If the participant did not receive a call from the WASHmobile mHealth program (missed or call failed), a subsequent call was sent after 15 min, 30 min, 1 h, and 24 h. Messages were sent to all mobile phones in the household, and phone owners were encouraged to share messages with other household members.

The WASHmobile PICHA7 program was developed through the previous work conducted in Bangladesh and community-centered formative research conducted in DRC. This formative research in the DRC included semi-structured interviews with program participants and a pilot study of 518 participants [21]. The mHealth messages were developed and tailored during formative research.

This study focuses specifically on the mHealth component of the WASHmobile program.

### EngageSPARK platform process evaluations indicators

Study process evaluation indicators are presented using the Medical Research Council framework for process evaluation [22]. The process evaluation indicators assessed using the engageSPARK platform were the following: (1) the percentage of WASHmobile voice, IVR, and text messages received by program households (fidelity, targeted to at least 80%); (2) the percentage of voice, IVR, and text messages answered (voice and IVR received) and fully listened to (voice and IVR) by program households (dose); (3) the percentage of program households replying correctly to WASHmobile IVR quiz responses and (4) the percentage of program households that reported receiving a WASHmobile mHealth message in the past 2 weeks (reach). The percentage of WASHmobile unique text messages received by program households was calculated by dividing the total number of unique text messages received by the total number of unique text messages sent. The percentage of unique voice and IVR messages answered by program households was calculated by dividing the total number of unique messages answered by the total unique number of messages sent to the households. The engageSPARK platform was also used to determine how long (call duration) a recorded voice and IVR message was listened to before the call ended. If a voice or IVR message was answered, it was classified as “fully listened” if at least 80% of the message was completed (based on the length of the full recorded message); otherwise, this was classified as “partially listened”. The percentage of PICHA7 unique voice and IVR messages fully listened to by PICHA7 mHealth program households was calculated by dividing the number of unique messages fully listened to by the total number of unique messages answered.

For IVR messages, response options for a question were pressing either 1 or 2 based on which statement the participant thought was correct. When the respondent selected a response, the answer was classified as “replied” and given the status of “correct” or “incorrect” (pressed 1 or 2), or “invalid” (not 1 or 2 (e.g., 3)). The percentage of IVR messages replied correctly was calculated by dividing the total number of IVR messages replied “correct” by the total number of IVR messages replied to.

### Data collection for reported mHealth program interaction

Participants 12 years of age or older were administered monthly in-person questionnaires to assess their interactions with the mHealth program. Program reach was assessed using the percentage of households and participants that reported receiving or sharing a WASHmobile mHealth message in the past 2 weeks. Information was also collected on the percentage of individuals in program households that reported challenges with receiving WASHmobile mHealth messages during the program period. The percentage of households and participants that reported interacting with a WASHmobile mHealth message in the past two weeks was calculated by dividing the number of households and participants (≥ 12 years) that reported receiving a mHealth message in the past 2 weeks by the total number of households and participants (≥ 12 years) with monthly surveillance data available at each time point.

### Data collection for diarrhea, handwashing, and water *E. coli* outcomes

During the study period, we conducted monthly clinical surveillance of diarrhea (3 or more loose stools in a 24 h period in the past 2 weeks) among all diarrhea patient household members (all age groups). Unannounced spot check visits were conducted on Day 7 and Months 1, 3, 6, 9, and 12 after enrollment in a randomly selected subset of 100 households per study arm for water sample collection (storage water and water source) for the *E. coli* analysis. Five-hour structured observation was conducted on Day 7 and Months 1, 3, 6, 9, and 12 after enrollment in a randomly selected subset of 50 households per study arm to observe handwashing with soap behavior at key events. Handwashing with soap was defined as washing both hands with a cleansing agent (bar soap, detergent powder, liquid soap, ash, or soapy water). Key events for handwashing recorded during structured observation include (1) before food preparation (food event); (2) before eating (food event); (3) before feeding someone (food event); (4) before serving food (food event); (5) after going to the toilet (stool event); (6) after disposing of stool (stool event); and (7) after washing the anus of children (stool event).

We report the results of the behavioral outcomes in George et al. 2025. We found that delivery of the WASHmobile PICHA7 program significantly increased handwashing with soap at food and stool-related events and chlorine being present in stored water during and reduced *E. coli* in stored drinking water, which was sustained to the 12-month follow-up [20].

### Statistical analysis

Descriptive statistics were used to define the study population. We used logistic regression to assess the impact of IVR quiz responses on diarrhea, handwashing with soap, and household stored water *E. coli* concentration at the subsequent month. Predictors were: (1) correct answer for IVR message response vs. did not answer and (2) answered IVR message versus did not answer. Diarrhea, handwashing with soap, and household stored water *E. coli* concentration at the subsequent month (next month) were the outcomes and were collected between 20 and 40 days after the IVR message was sent. All regressions were performed using generalized estimating equations to account for clustering at the household and participant levels. Analyses were completed using SAS version 9.4.

### Ethical approval

The WASHmobile PICHA7 research program was approved and validated by the ethics committee of the Johns Hopkins University (JHU), Bloomberg School of Public Health (9848), and the Catholic University of Bukavu (UCB) (7107). Informed consent was received from all participants or guardians.

## Results

Overall, 1196 participants were enrolled in the WASHmobile PICHA7 program (Table 1). There were 475 participants over 12 years of age, of whom 71% (335/475) were over 18 years of age. The mean age for WASHmobile participants was 27 years, and 68% (321/475) of participants were female. Ninety-nine percent (458/462) of participants had at least one household member who could read and write, and 5% (22/462) of participants reported refrigerator ownership. The average number of mobile phones per household enrolled in the WASHmobile program at baseline was 1 ± 0.7 (standard deviation) (range 1–5), and the average household size was 8.0 ± 2.7 [2–16]. For reported phone ownership, 52% (833/1601) of women reported access to a phone in the past 2 weeks compared to 40% (193/480) of men (chi-square p-value = 0.0001).

**Table 1:** Baseline WASHmobile PICHA7 participant characteristics

	%	*n*	*N*
Households			180
Participants			1196
Participants ≥ 12 years of age			475
Participants < 5 years of age			406
Primary caregivers			251
Mean ± SD (min–max)	1.5 ± 0.9 (1–7)		
Household size			170
Mean ± SD (min–max)	8.0 ± 2.7 (1–15)		
^§^Age (years)			
Mean ± SD (min–max)	27.1 ± 12.7 (12–79)		
12–18	29%	140	475
≥ 18	71%	335	475
^§^Gender			
Female	68%	321	475
Phones per household			
Mean ± SD (min–max)	1.0 ± 0.7 (1–5)		
^§^Household roof type			
Concrete	5%	24	462
Other	95%	438	462
^§^Household income			
Household income	96%	441	457
No household income	4%	16	457
^§^Refrigerator ownership	5%	22	462
^§^At least one household member who can read and write	99%	458	462

SD = standard deviation

§ Among participants ≥ 12 years of age. Participants ≥ 12 years of age assessed for interaction with mHealth program and represent primary group of interest for analysis

All primary caregivers ≥ 12 years of age

Household characteristics (household roof type, household income (measured as the household reporting any current income source vs. no current income source), refrigerator ownership, reading and writing information) reported at the participant level

Eighty-four percent of unique text messages sent to PICHA7 program households (15,132/18,042) during the 12-month program period were received (Table 2). Ninety percent (8122/9046) of unique voice calls were answered by program households, and 86% (7021/8122) of these voice calls were listened to fully. Ninety percent (8394/9298) of unique IVR calls were answered by program households, and 78% (6542/8394) of these IVR calls were fully listened to. All households that received an IVR message replied to at least one IVR message during the study period. Overall, 60% (2372/3985) of unique replies were correct, and 22% (884/3985) of unique replies were invalid. A full summary of household responses to unique IVR quiz questions is found in Additional File 2.

**Table 2:** Summary of WASHmobile PICHA7 program mobile messages sent to households

		All messages		Unique messages	
Message type	Indicator	*n*	%	*n*	%
Text	Sent	38,467	–	18,042	–
	Not delivered	11,390	30	2910	16
	Delivered	27,077	70	15,132	84
Voice	Sent	66,480	–	9046	–
	Not delivered	926	1	33	< 1
	No answer	50,379	76	891	10
	Answered	15,175	23	8122	90
	Partially listened	3463	23	1101	14
	Fully listened^†^	11,712	77	7021	86
Interactive voice response	Sent	64,048	–	9298	–
	Not delivered	926	1	32	< 1
	No answer	48,074	75	872	9
	Answered	15,048	23	8394	90
	Partially listened	4865	32	1852	22
	Fully listened^†^	10,183	68	6542	78

Unique messages indicate the number of unique messages sent to 180 households, and if at least one of each unique message was delivered/answered/listened to fully listened indicates at least 80% of the message was completed before the call was ended

**Fig. 2:**
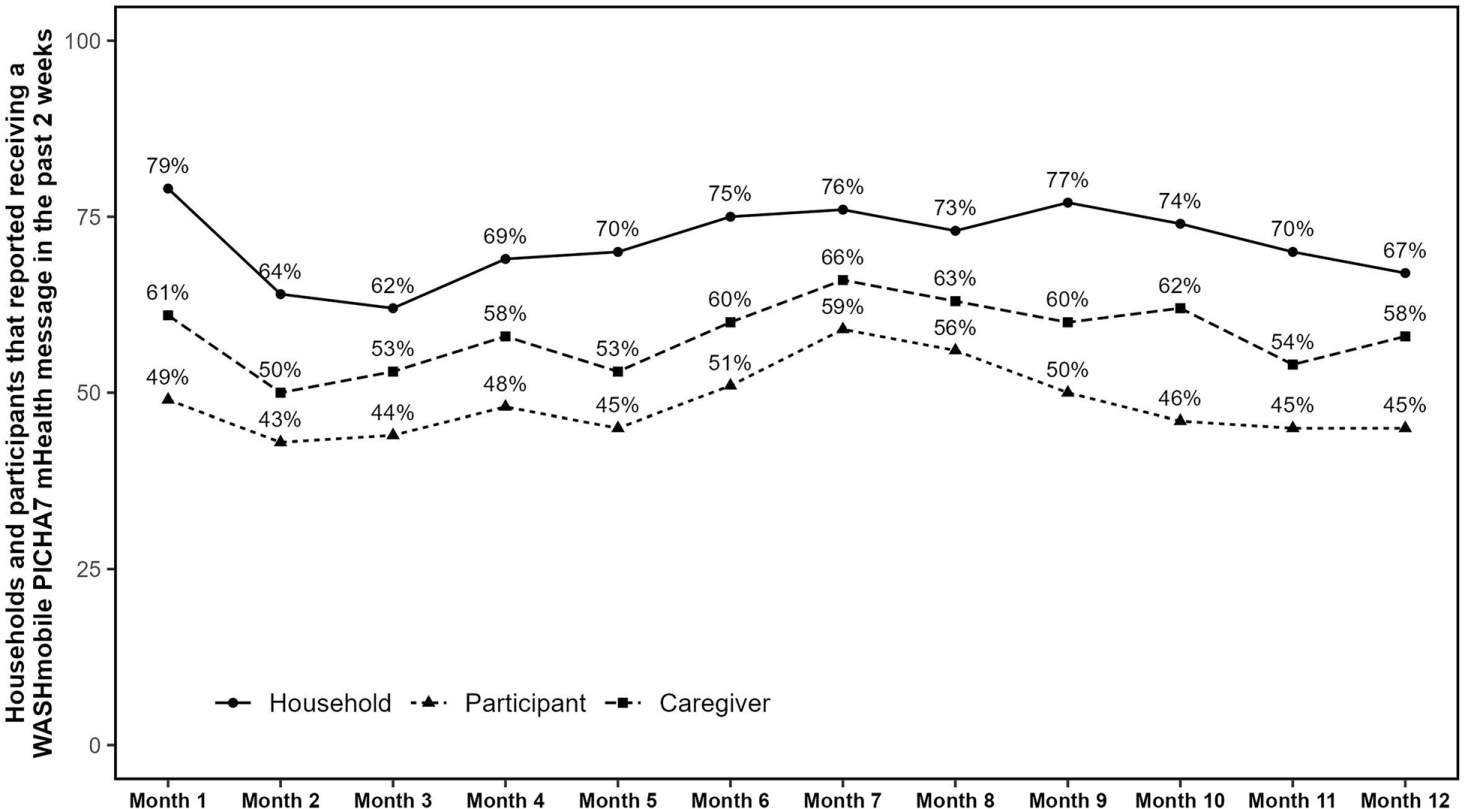
WASHmobile PICHA7 participant and households reporting receiving an mHealth message in the previous 2 weeks. Only includes participants age 12 or over. Household visits = 1177, Participant visits = 2024, Caregiver visits = 1051

WASHmobile household members reported receiving a program mHealth message in the past 2 weeks at 72% (844/1177) of surveillance visits (Fig. 2). Seventy-three percent (309/422) of participants reported sharing a WASHmobile message with another person at least once during the study period (Table 3). Phone access was significantly higher among females, with 52% of women reporting access to a phone in the past 2 weeks compared to 40% of men (chi-square *p*-value = 0.0001). Twenty-three percent (59/259) of participants reported that distracting or noisy background sounds made it hard to hear voice calls during the program period (Table 4), 22% (58/258) of participants reported that they were unclear on how to reply to an IVR message, 21% (56/270) reported that their phone was damaged, and 20% (53/261) reported that poor mobile phone reception made a voice/IVR call hard to hear. No significant association was found between replying to an IVR message and diarrhea prevalence, handwashing with soap, or household stored water *E. coli* concentration (Table 5).

**Table 3:** Participants reporting sharing a WASHmobile PICHA7 mHealth message with others and those reporting someone shared a message with them during the study period (*N* = 422)

	You shared with % (*n*)	Someone shared with you % (*n*)
Any sharing	73% (309)	64% (270)
Spouse	27% (113)	38% (159)
Neighbor	5% (20)	44% (187)
Children	20% (84)	62% (261)
Parents	31% (131)	32% (136)
Grandparents	3% (12)	9% (39)
Sibling	16% (68)	43% (180)
In-law	3% (11)	7% (31)
Friends	6% (25)	46% (193)
Co-worker	1% (6)	11% (48)
Other	10% (41)	5% (19)

More than 1 response possible per participant at each timepoint

Sharing messages in the past 2 weeks (month 1 visit and later)

*N* = total number of responses over study period

Only includes participants age 12 or over

**Table 4:** Reported challenges with the WASHmobile PICHA7 program over the study period (*N* = 416)

Challenge	%
Distracting/noisy background made it hard to hear the voice call	23
Unclear on how to reply to quiz (question where we asked you to press 1 or 2) questions	23
Phone is damaged	21
Poor cell phone reception made voice call hard to hear	20
Not enough messages received—need more guidance	17
Accidentally hung up on voice call before message finished	15
Message arrived at time inconvenient for reading the text or listening to the voice call	14
When you answer the call it is cut by the sender after a few seconds	14
Phone is Lost	14
Too busy to receive messages	13
Did not have time to listen to full voice call	12
Accidentally deleted SMS before reading it	12
Too many messages received—burdensome	11
No one available to read the SMS	10
Message not shared by phone owner	9
Afraid to reply to quizzes (question where we asked you to press 1 or 2) because might be charged	9
Full inbox blocked incoming messages	9
You are concerned that if you press the wrong answer something bad will happen	8
You cannot read the SMS message	5
Instructions in the messages are confusing	4
Message content not interesting or pertinent to needs	3
Other	26

More than 1 response possible per participant

Challenges in the past 2 weeks assessed during monthly visits

*N* = total number of overall participants

Only includes participants age 12 or over

**Table 5:** Association between a response to a WASHmobile PICHA7 IVR quiz message, diarrhea in the previous 2 weeks, and WASH behaviors at subsequent month household visit

Group	Correct answer for IVR message response (vs. did not answer)					Answered IVR message (vs. did not answer)				
	Correct answer		Did not answer			Answered		Did not answer		
	%	N	%	N	OR (95% CI)	%	N	%	N	OR (95% CI)
	% diarrhea in past 2 weeks					% diarrhea in past 2 weeks				
Diarrhea										
All ages	7%	3101	7%	1572	0.92 (0.7, 1.22)	7%	4282	7%	1572	0.86 (0.66, 1.12)
Children < 5 years	12%	1198	13%	602	1.02 (0.74, 1.4)	12%	194	13%	602	0.96 (0.71, 1.3)
Children < 2 years	16%	569	19%	309	0.91 (0.64, 1.3)	16%	779	19%	309	0.88 (0.63, 1.22)
Handwashing with soap										
Any event	49%	618	49%	293	0.85 (0.59, 1.23)	49%	851	49%	293	0.9 (0.64, 1.26)
Food event	45%	552	47%	256	0.89 (0.57, 1.38)	44%	731	47%	256	0.93 (0.62, 1.39)
Stool event	45%	303	40%	139	0.95 (0.61, 1.48)	47%	407	40%	139	0.99 (0.67, 1.45)
Household stored water *E. coli* concentration										
< 1/100 mL CFU	90%	227	89%	104	1.22 (0.57, 2.58)	89%	303	89%	104	1.04 (0.54, 2.01)
< 100/100 mL CFU	98%	227	96%	104	1.8 (0.47, 6.94)	89%	303	96%	104	1.7 (0.48, 6.02)
< 1000/100 mL CFU	99%	227	100%	104	–	99%	303	100%	104	–

Handwashing for participants age 2 or greater

OR = Odds ratio

CI = Confidence interval

Diarrhea prevalence is from monthly clinical surveillance of diarrhea (3 or more loose stools in a 24-h period in the past 2 weeks). Unannounced spot check visits were conducted at Day 7 and 1, 3, 6, 9, and 12 months after enrollment in a randomly selected subset of 100 households per study arm for water sample collection for the *E. coli* analysis. Five-hour structured observation was conducted at Day 7 and 1, 3, 6, 9, and 12 months after enrollment in a randomly selected subset of 50 households per study arm to observe handwashing with soap behavior at key events. Handwashing with soap was defined as washing both hands with a cleansing agent (bar soap, liquid soap, ash or soapy water). Key events for handwashing recorded during structured observation include (1) before food preparation (food event), (2) before eating (food event), (3) before feeding someone (food event), (4) before serving food (food event), (5) after going to the toilet (stool event), (6) after disposing of stool (stool event), and (7) after washing the anus of children (stool event)

## Discussion

The process evaluation of the WASHmobile PICHA7 program demonstrated high fidelity, dose, and reach of program calls and text messages to households. Ninety percent of unique voice and IVR calls were answered and over 80% of text messages were received by program households, with the majority of unique voice calls (86%) and IVR calls (78%) being fully listened to. Furthermore, program households reported receiving a WASHmobile message in the past 2 weeks at the majority of surveillance visits (72%). A high proportion of message sharing was also observed among those receiving program mobile messages (73%). These results demonstrate that the WASHmobile PICHA7 program presents a promising approach to deliver voice and IVR calls and text messages in this study setting to reinforce the WASH behaviors promoted during in-person visits for program delivery. The high program fidelity, dose, and reach complement our RCT of the WASHmobile PICHA7 mHealth program which showed that when combined with quarterly in-person visits, the mHealth program was effective in significantly increasing handwashing with soap and water treatment behaviors and lowering healthcare facility visits for diarrhea and stunting during the 12-month program compared to those who did not receive the program [20].

The success of the WASHmobile PICHA7 program in terms of fidelity, dose, and reach is likely attributed to the community-centered formative research conducted over an 18-month period prior to the implementation of the program [21]. This formative research allowed us to tailor the WASHmobile program to address identified barriers to effective implementation. For example, during the pilot study conducted as part of this formative research, we identified that participants had difficulty understanding some voice and IVR messages. In response, we simplified the language in these messages to accommodate those with limited or no formal education before implementing the RCT program. During the pilot, participants also requested that voice and IVR calls be sent in the evenings between 7 and 9 PM instead of between 4 and 6 PM, to ensure most household members were home and could listen to messages together. In addition, the pilot highlighted the importance of an initial WASHmobile mHealth program orientation at the healthcare facility, where a practice voice call, IVR call, and summary text were sent to each phone of diarrhea patient household members as part of hands-on training. This training allowed for major technical challenges with message delivery to be addressed at the start of the program. Future WASH mHealth programs should similarly engage community members in intervention development and delivery and conduct pilot studies prior to large-scale program implementation.

Our findings are consistent with our previous WASHmobile CHoBI7 WASH mHealth program in Bangladesh, which also demonstrated high fidelity, dose, and reach in program delivery to diarrhea patient households [23]. In the WASHmobile CHoBI7 mHealth program, 83% of voice calls and 86% of IVR calls were fully listened to and 92% of text messages were received by program households. At the 12-month timepoint, 78% of program households reported receiving a WASHmobile CHoBI7 mHealth message in the past 2 weeks during surveillance visits. This program was also developed through community-centered formative research [16]. Consistent with WASHmobile PICHA7, the CHoBI7 mHealth program combined with in-person visits significantly increased handwashing with soap and water treatment behaviors while also reducing diarrhea prevalence and stunting. These findings demonstrate that WASHmobile which  combines mHealth with in-person visits can reduce diarrhea and improve child growth in diarrhea patient households in two very distinct contexts,  the DRC and Bangladesh.

The delivery of the WASHmobile PICHA7 program encountered some implementation challenges. These included background noise making calls hard to hear, participants being unclear on how to reply to IVR calls, household members having damaged phones, and poor cell phone reception. Similar challenges have been reported in other mHealth studies conducted in Africa, where participants also faced difficulties related to changing phone numbers, limited time for intervention interactions, and technical issues (e.g., only receiving part of messages or receiving messages in two parts) [24, 25]. Low message response rates and difficulties with IVR message replies have also been reported in other studies in sub-Saharan Africa [24, 26, 27]. However, despite these challenges, mHealth programs in Africa have generally been found to be acceptable and capable of achieving desired outcomes. For example, one study in South Africa on a preconception health trial found higher self-reported SMS text intervention adherence and better hemoglobin levels among those that received a 2-way text intervention compared to those that did not; however, there was only an 11% message response rate [24].

We found no significant association between interacting with IVR messages and diarrhea prevalence, handwashing with soap, or household stored water *E. coli* concentration. This finding indicates that there may be no significant added benefit to engagement with the IVR component of the intervention (responding to IVR messages), and that engagement of the WASHmobile program through voice, text, and quarterly in-person visits alone may be sufficient to increase WASH behaviors and reduce diarrhea diseases. One possible explanation is that technical challenges with the ability of users to understand how to respond to IVR messages limited usage of this feature, even as participants were listening to and understanding the IVR message content. Interestingly, we found that phone access was significantly higher among female household members, with 52% of women reporting access to a phone in the past 2 weeks compared to 40% of men (chi-square p-value = 0.0001). This was an unexpected finding and likely contributed to the success of the WASHmobile PICHA7 program through penetration of this program with a traditionally harder-to-reach population for mHealth programs in low-income countries such as the DRC.

This study has some limitations. First, this study  was conducted in an urban area and therefore findings cannot be generalized to rural areas. Future studies should pilot the WASHmobile program in rural areas of DRC. Second, our PICHA7 program was focused on diarrhea patient households which reported having at least one functioning mobile phone in their home. Future studies should include households that have shared access to phones with other households. Additionally, voice, IVR, and text messages were only provided in Swahili, the primary language in our study setting. Future programs should include multiple language options at sign-up to reach a broader audience.

This study has strengths. The first strength is the monthly surveillance of the mHealth process evaluation indicators which allowed us to investigate program reach and challenges program recipients faced. Second, the use of the process evaluation indicators from the engageSPARK platform (fidelity and dose) to assess call and message status (received, answered, full listened and key pressed for quiz messages) was a major strength. Finally, the investigation of the role of program household IVR responses on WASH and health outcomes.

## Conclusion

The WASHmobile PICHA7 program had high fidelity, dose, and reach for voice and IVR calls and text messages sent to program households. These findings demonstrate the feasibility of delivering the WASHmobile program in our study setting in eastern DRC and provide important insights for developing WASH mHealth programing in low- and middle-income countries globally. The WASHmobile program combining mHealth messages and in-person visits is a promising approach to increase WASH behaviors and reduce diarrhea and stunting in DRC.

## Supplementary Information

Additional file1 (PDF 501 kb)

Additional file2 (PDF 128 kb)

## Data Availability

The datasets analyzed during the current study are available from the corresponding author on request.

## Abbreviations

DRC: Democratic Republic of the Congo
WASH: Water, sanitation, and hygiene
mHealth: Mobile health
PICHA7: Preventative-Intervention-for-Cholera-for-7-days
RCT: Randomized control trial
IVR: Interactive voice response
CHoBI7: Cholera-Hospital-Based-Intervention-for-7-Days
ORS: Oral rehydration solution
JHU: Johns Hopkins University
UCB: Catholic University of Bukavu
